# Supplementary material for: Why does the X chromosome lag behind autosomes in GWAS findings?
Source: PLoS Genet. 2023 Feb 27;19(2):e1010472. doi: 10.1371/journal.pgen.1010472 (PMC9997976; doi:10.1371/journal.pgen.1010472)
Supplement: S5 Table — (DOCX) [file pgen.1010472.s005.docx]

**Genome wide association studies randomly selected to find if they mentioned including males in HWE testing.**

1. Balakrishnan P, Vaidya D, Voruganti VS, Haack K, Kent JW, North KE, et al. Genetic Variants Related to Cardiometabolic Traits Are Associated to B Cell Function, Insulin Resistance, and Diabetes Among AmeriCan Indians: The Strong Heart Family Study. Front Genet. 2018;9:466. Epub 2018/10/30. doi: 10.3389/fgene.2018.00466. PubMed PMID: 30369944; PubMed Central PMCID: PMCPMC6194194.

2. Baldassari AR, Sitlani CM, Highland HM, Arking DE, Buyske S, Darbar D, et al. Multi-Ethnic Genome-Wide Association Study of Decomposed Cardioelectric Phenotypes Illustrates Strategies to Identify and Characterize Evidence of Shared Genetic Effects for Complex Traits. Circ Genom Precis Med. 2020;13(4):e002680. Epub 2020/07/01. doi: 10.1161/CIRCGEN.119.002680. PubMed PMID: 32602732; PubMed Central PMCID: PMCPMC7520945.

3. Baselmans BML, Jansen R, Ip HF, van Dongen J, Abdellaoui A, van de Weijer MP, et al. Multivariate genome-wide analyses of the well-being spectrum. Nat Genet. 2019;51(3):445-51. Epub 2019/01/16. doi: 10.1038/s41588-018-0320-8. PubMed PMID: 30643256.

4. Casto AM, Seo S, Levine DM, Storer BE, Dong X, Hansen JA, et al. Genetic variants associated with cytomegalovirus infection after allogeneic hematopoietic cell transplantation. Blood. 2021;138(17):1628-36. Epub 2021/07/17. doi: 10.1182/blood.2021012153. PubMed PMID: 34269803; PubMed Central PMCID: PMCPMC8554648.

5. Choquet H, Melles RB, Yin J, Hoffmann TJ, Thai KK, Kvale MN, et al. A multiethnic genome-wide analysis of 44,039 individuals identifies 41 new loci associated with central corneal thickness. Commun Biol. 2020;3(1):301. Epub 2020/06/13. doi: 10.1038/s42003-020-1037-7. PubMed PMID: 32528159; PubMed Central PMCID: PMCPMC7289804.

6. Dahlin A, Sordillo JE, McGeachie M, Kelly RS, Tantisira KG, Lutz SM, et al. Genome-wide interaction study reveals age-dependent determinants of responsiveness to inhaled corticosteroids in individuals with asthma. PLoS One. 2020;15(3):e0229241. Epub 2020/03/03. doi: 10.1371/journal.pone.0229241. PubMed PMID: 32119686; PubMed Central PMCID: PMCPMC7051058.

7. Du M, Garcia JGN, Christie JD, Xin J, Cai G, Meyer NJ, et al. Integrative omics provide biological and clinical insights into acute respiratory distress syndrome. Intensive Care Med. 2021;47(7):761-71. Epub 2021/05/26. doi: 10.1007/s00134-021-06410-5. PubMed PMID: 34032881; PubMed Central PMCID: PMCPMC8144871.

8. Eckel-Passow JE, Drucker KL, Kollmeyer TM, Kosel ML, Decker PA, Molinaro AM, et al. Adult diffuse glioma GWAS by molecular subtype identifies variants in D2HGDH and FAM20C. Neuro Oncol. 2020;22(11):1602-13. Epub 2020/05/10. doi: 10.1093/neuonc/noaa117. PubMed PMID: 32386320; PubMed Central PMCID: PMCPMC7690366.

9. El Rouby N, McDonough CW, Gong Y, McClure LA, Mitchell BD, Horenstein RB, et al. Genome-wide association analysis of common genetic variants of resistant hypertension. Pharmacogenomics J. 2019;19(3):295-304. Epub 2018/09/22. doi: 10.1038/s41397-018-0049-x. PubMed PMID: 30237584; PubMed Central PMCID: PMCPMC6426691.

10. Guo W, Machado-Vieira R, Mathew S, Murrough JW, Charney DS, Grunebaum M, et al. Exploratory genome-wide association analysis of response to ketamine and a polygenic analysis of response to scopolamine in depression. Transl Psychiatry. 2018;8(1):280. Epub 2018/12/16. doi: 10.1038/s41398-018-0311-7. PubMed PMID: 30552317; PubMed Central PMCID: PMCPMC6294748 the National Institute of Mental Health, National Institutes of Health (IRP-NIMH-NIH; ZIA MH002857, ZIA MH002930), by a NARSAD Independent Investigator Award to C.A.Z., and by a Brain and Behavior Mood Disorders Research Award to C.A.Z. C.A.Z. is listed as a coinventor on a patent for the use of ketamine and its metabolites in major depression and suicidal ideation. C.A.Z. is listed as a coinventor on a patent for the use of (2 R,6 R)-hydroxynorketamine, (S)-dehydronorketamine, and other stereoisomeric dehydro and hydroxylated metabolites of (R,S)-ketamine metabolites in the treatment of depression and neuropathic pain. C.A.Z. is listed as coinventor on a patent application for the use of (2 R,6 R)-hydroxynorketamine and (2 S,6 S)-hydroxynorketamine in the treatment of depression, anxiety, anhedonia, suicidal ideation, and post-traumatic stress disorders; he has assigned his patent rights to the US government but will share a percentage of any royalties that may be received by the government. J.J.M. and M.A.O. receive royalties for commercial use of the C-SSRS from the Research Foundation for Mental Hygiene. M.A.O.'s family owns stock in Bristol Myers Squibb. In the past three years, J.W.M. has provided consultation services for Fortress Biotech, Inc., Novartis, Janssen Research and Development, Genentech, and ProPhase, and has received research support from Avanir Pharmaceuticals, Inc. J.W.M. is named on a pending patent for lithium to extend the antidepressant effect of ketamine and for the combination of lithium and ketamine for the treatment of suicidal ideation. The Icahn School of Medicine at Mount Sinai (with which J.W.M. is affiliated) is named on a patent, has entered into a licensing agreement, and will receive payments related to the use of ketamine if it is approved for the treatment of depression. J.W.M. is not named on the patent and will not receive any payments. M.F. and W.D. are employees of Janssen Pharmaceuticals and have patent on scopolamine. All remaining authors have no conflicts of interest to disclose, financial or otherwise.

11. Hu Y, Bien SA, Nishimura KK, Haessler J, Hodonsky CJ, Baldassari AR, et al. Multi-ethnic genome-wide association analyses of white blood cell and platelet traits in the Population Architecture using Genomics and Epidemiology (PAGE) study. BMC Genomics. 2021;22(1):432. Epub 2021/06/11. doi: 10.1186/s12864-021-07745-5. PubMed PMID: 34107879; PubMed Central PMCID: PMCPMC8191001.

12. Knol MJ, Lu D, Traylor M, Adams HHH, Romero JRJ, Smith AV, et al. Association of common genetic variants with brain microbleeds: A genome-wide association study. Neurology. 2020;95(24):e3331-e43. Epub 2020/09/12. doi: 10.1212/WNL.0000000000010852. PubMed PMID: 32913026; PubMed Central PMCID: PMCPMC7836652.

13. Liu M, Jiang Y, Wedow R, Li Y, Brazel DM, Chen F, et al. Association studies of up to 1.2 million individuals yield new insights into the genetic etiology of tobacco and alcohol use. Nat Genet. 2019;51(2):237-44. Epub 2019/01/16. doi: 10.1038/s41588-018-0307-5. PubMed PMID: 30643251; PubMed Central PMCID: PMCPMC6358542.

14. Liyanage UE, Law MH, Han X, An J, Ong JS, Gharahkhani P, et al. Combined analysis of keratinocyte cancers identifies novel genome-wide loci. Hum Mol Genet. 2019;28(18):3148-60. Epub 2019/06/08. doi: 10.1093/hmg/ddz121. PubMed PMID: 31174203; PubMed Central PMCID: PMCPMC6737293.

15. Mullins N, Bigdeli TB, Borglum AD, Coleman JRI, Demontis D, Mehta D, et al. GWAS of Suicide Attempt in Psychiatric Disorders and Association With Major Depression Polygenic Risk Scores. Am J Psychiatry. 2019;176(8):651-60. Epub 2019/06/06. doi: 10.1176/appi.ajp.2019.18080957. PubMed PMID: 31164008; PubMed Central PMCID: PMCPMC6675659.

16. Pilling LC, Atkins JL, Duff MO, Beaumont RN, Jones SE, Tyrrell J, et al. Red blood cell distribution width: Genetic evidence for aging pathways in 116,666 volunteers. PLoS One. 2017;12(9):e0185083. Epub 2017/09/29. doi: 10.1371/journal.pone.0185083. PubMed PMID: 28957414; PubMed Central PMCID: PMCPMC5619771.

17. Pilling LC, Kuo CL, Sicinski K, Tamosauskaite J, Kuchel GA, Harries LW, et al. Human longevity: 25 genetic loci associated in 389,166 UK biobank participants. Aging (Albany NY). 2017;9(12):2504-20. Epub 2017/12/12. doi: 10.18632/aging.101334. PubMed PMID: 29227965; PubMed Central PMCID: PMCPMC5764389.

18. Pott J, Beutner F, Horn K, Kirsten H, Olischer K, Wirkner K, et al. Genome-wide analysis of carotid plaque burden suggests a role of IL5 in men. PLoS One. 2020;15(5):e0233728. Epub 2020/05/30. doi: 10.1371/journal.pone.0233728. PubMed PMID: 32469969; PubMed Central PMCID: PMCPMC7259763.

19. Truong DT, Adams AK, Paniagua S, Frijters JC, Boada R, Hill DE, et al. Multivariate genome-wide association study of rapid automatised naming and rapid alternating stimulus in Hispanic American and African-American youth. J Med Genet. 2019;56(8):557-66. Epub 2019/04/19. doi: 10.1136/jmedgenet-2018-105874. PubMed PMID: 30995994; PubMed Central PMCID: PMCPMC6678051.

20. Wang TM, Shen GP, Chen MY, Zhang JB, Sun Y, He J, et al. Genome-Wide Association Study of Susceptibility Loci for Radiation-Induced Brain Injury. J Natl Cancer Inst. 2019;111(6):620-8. Epub 2018/10/10. doi: 10.1093/jnci/djy150. PubMed PMID: 30299488; PubMed Central PMCID: PMCPMC6579742.

21. Ware EB, Smith JA, Zhao W, Ganesvoort RT, Curhan GC, Pollak M, et al. Genome-wide Association Study of 24-Hour Urinary Excretion of Calcium, Magnesium, and Uric Acid. Mayo Clin Proc Innov Qual Outcomes. 2019;3(4):448-60. Epub 2020/01/30. doi: 10.1016/j.mayocpiqo.2019.08.007. PubMed PMID: 31993563; PubMed Central PMCID: PMCPMC6978610.

22. Wei J, Sheng Y, Li J, Gao X, Ren N, Dong Q, et al. Genome-Wide Association Study Identifies a Genetic Prediction Model for Postoperative Survival in Patients with Hepatocellular Carcinoma. Med Sci Monit. 2019;25:2452-78. Epub 2019/04/05. doi: 10.12659/MSM.915511. PubMed PMID: 30945699; PubMed Central PMCID: PMCPMC6461006.

23. Winkler TW, Grassmann F, Brandl C, Kiel C, Gunther F, Strunz T, et al. Genome-wide association meta-analysis for early age-related macular degeneration highlights novel loci and insights for advanced disease. BMC Med Genomics. 2020;13(1):120. Epub 2020/08/28. doi: 10.1186/s12920-020-00760-7. PubMed PMID: 32843070; PubMed Central PMCID: PMCPMC7449002.

24. Yao X, Glessner JT, Li J, Qi X, Hou X, Zhu C, et al. Integrative analysis of genome-wide association studies identifies novel loci associated with neuropsychiatric disorders. Transl Psychiatry. 2021;11(1):69. Epub 2021/01/23. doi: 10.1038/s41398-020-01195-5. PubMed PMID: 33479212; PubMed Central PMCID: PMCPMC7820351.

25. Yap CX, Sidorenko J, Wu Y, Kemper KE, Yang J, Wray NR, et al. Dissection of genetic variation and evidence for pleiotropy in male pattern baldness. Nat Commun. 2018;9(1):5407. Epub 2018/12/24. doi: 10.1038/s41467-018-07862-y. PubMed PMID: 30573740; PubMed Central PMCID: PMCPMC6302097.
