# Supplementary material for: Why does the X chromosome lag behind autosomes in GWAS findings?
Source: PLoS Genet. 2023 Feb 27;19(2):e1010472. doi: 10.1371/journal.pgen.1010472 (PMC9997976; doi:10.1371/journal.pgen.1010472)
Supplement: Attachment — Submitted filename: Response to Reviwers.docx [file pgen.1010472.s007.docx]

Dear Drs. Ziegler and Williams,

We are resubmitting a revised paper, '**Why does the X chromosome lag behind autosomes in GWAS findings?**' The previous title of the manuscript was 'Why are GWASs limp about the X chromosome?' Hereafter we provide point-by-point replies to the Reviewers’ comments. All corrections in the revised manuscript are shown in red.

***Reviewer #1:*** *The authors "found that the density of GWAS-detected SNPs on X-chromosome is almost 6-fold lower compared to that on autosomes. Analysis stratified by SNP location in transcribed and intergenic regions supported the idea that the lower density of GWAS-detected SNPs on X-chromosome is a result of a stronger selection against functional SNPs on X-chromosome." I quote this synopsis from the manuscript not only to summarize the work but also to show the authors' crisp and informative language. The authors present a compelling case that few X-linked SNPs have been identified as being associated with traits in GWAS studies. Their datasets are large (1000 Genomes, TOPMed and the Catalog of Published GWASs) and their statistical analyses are simple but appropriate.*

*The authors state "The recommended approach to deal with this issue is to run HWE test only in females [21, 22]." This seems to contradict reference [21] where a test that combines HWE testing and male-female allele-frequency difference testing was discussed. The authors could confine their analyses here to female-only studies, but they could report on the rates of HWE rejection on autosomes vs X-chromosome.*

We revised the paragraph the Reviewer cited, as follows: "Initially recommended approach to deal with this issue is to run HWE test only in females [1-3]. More recently exact statistical test based on conditional distribution of the number of heterozygotes given the minor allele count has been proposed [4]. The test takes both male and female genotypes into account. For some SNPs the results from female-restricted HWE testing versus all-individuals-included testing are different, but overall the differences are not dramatic (see figure 7 in [4].)”

We added the following sentence to the Discussion: “Based on available publications [5, 6] and our analysis (Fig. 6), call rates for X-chromosome SNPs are similar to call rates for autosomal SNPs.”

*The authors focus on GWAS-detected SNPs. What do they conclude about findings from other methods, such as linkage studies? Would those not also be affected by selection against X-variants?*

We focused on the analysis of GWAS-detected phenotype/disease associated SNPs because for over a decade, GWA studies have been the prime method to uncover genetic control of phenotypic variation. GWASs generated a plethora of the data allowing their reliable statistical exploration.

We found that a dearth of GWAS hits on X-chromosome compared to autosomes is likely to be a result of stronger negative selection against functional variants on X-chromosome compared to autosomes because X-chromosome SNPs are more “open” to selection due to hemizygosity in males and X-chromosome inactivation in females. We think that any method reliably detecting genotype/phenotype association would show a similar difference between X-chromosome and autosome in the density of hits.

*The authors' "We tested all possible technical/methodological explanations of the X-chromosome versus autosomal differences in the density of GWAS-detected SNPs." should at least be modified to refer to explanations they can think of or have read about. It does not detract from their work to acknowledge there still may be other technical/methodological explanations. It doesn't appear that they looked at male-female allele frequency differences, for example. Have they ruled out possible bias in CPGWAS resulting from fewer reported GWAS that looked at X-chromosome SNPs?*

We softened the statement by replacing it with the following sentence: "We went through all technical/methodological explanations of the X-chromosome versus autosomal differences in the density of GWAS-detected SNPs of which we are aware."

To enhance selection-based biological explanation of the differences in the density of GWAS-detected SNPs between X-chromosome and autosomes we conducted a comparative analysis of segregating sites for missense mutations with different predicted functionality (please see responses to Reviewer 2 comments for more details).

***Reviewer #2****: This is an interesting manuscript which provides some data to suggest that the paucity of GWAS signals on X chromosome go beyond previously described phenomenon.

The title is potentially stigmatizing: specifically, the word limp has common sexual inferences: erectile dysfunction, limp-wristed, that I suspect the authors probably do not mean. An alternative word choice might be more appropriate.*

We changed the title to 'Why does the X chromosome lag behind autosomes in GWAS findings?'

*I think that the SNP consortium was the first to show reduced variation on X cf autosomes:* [*https://www.nature.com/articles/35057149*](https://www.nature.com/articles/35057149)

The following statement was added to Discussion: “Lower genetic variation on X-chromosome compared to autosomes was first reported by the SNP consortium [7] and later confirmed by other studies [8, 9].”

*Please specify which build of dbSNP was used. Similar for which release of TOPMed.*

We added build/release information for dbSNP and TOPMed.

*I don’t quite understand what was done for Fig 4. (Table S3). Did they look at each GWAS with X chrom signal, determine which platform was used, and then compute the # of X vs autosomal signals. Or did they just take all GWAS signals and see whether the SNP was on specific platforms?*

The goal of the Fig. 4 (Table S3) was to demonstrate that the difference between autosomal and X-chromosome SNPs in the density of GWAS hits remains when we consider SNPs that **were actually included in GWASs**. We retrieved lists of SNPs on each commonly used genotyping platform and among those SNPs identified those ever reported by a GWAS. To clarify this point we extended the description of how the analysis was conducted.

*Most GWASes now use imputation and meta-analysis where component studies have genotyped different arrays. How do these two aspects impact these findings? This is a major topic that is not addressed anywhere in the manuscript and could have implications for a number of their observations. In addition, commonly used references such as the 1000 Genomes phase 3 data has recently been shown to have sex differences in allele frequencies for some SNPs, which potentially could impact the results of analysis: PMID: 35639794.*

We added the following paragraph to the Discussion:

“Imputation is a commonly used approach to increase the number of SNPs analyzed in GWA studies [10, 11]. Based on the results of a recent study [12], imputation accuracy is essentially the same for autosomal and X-chromosome SNPs: 89.3% versus 90.2. This suggests that imputation accuracy is unlikely to be the source of observed conspicuous differences in GWAS hit densities between X-chromosome and autosomes.

The other analytical technique frequently used in GWA studies is meta-analysis [13, 14]. Meta-analysis improves the statistical power by combining statistics from several GWA studies. It is unlikely that this technique will contribute to X-chromosome versus autosomal differences in the GWAS hit densities since SNPs are included in meta-analysis regardless of their chromosomal location.

The other potential contributor to X-chromosome versus autosome differences in the density of GWAS findings is sex differences in allele frequencies. A recent study by Wang et al. [15] found that about 1% of SNPs on the X chromosome show sex differences in minor allele frequencies (MAFs). The study identified 2,039 SNPs with genome level differences in MAFs between males and females. We checked how many of those SNPs are reported by GWASs. We found that only one SNP from the list, rs4014653 was reported as a GWAS hit [16]. The frequency of GWAS-detected SNPs among SNPs with sex differences in MAF – 1/2039 is comparable with the fraction of GWAS-detected SNPs among all known SNPs (Fig. 2). We estimated how many SNPs with sex differences in MAFs are on commonly used on the Illumina Humanomni5-4v1_c platform. We found that 21 SNPs are on the platform; therefore, the frequency of GWAS hits among directly genotyped SNPs is 1/21=0.05 which is similar to the overall frequency of GWAS hits on the platform, 0.08 (Fig. 4). Thus it is unlikely that sex differences in MAFs is a major contributor to the deficit of X-chromosome SNPs in GWASs.”

*It is more conventional to refer to 1000 Genomes Project rather than ‘Thousand genomes’. Which version did they use? Seems to be phase 3. The latest data is high coverage WGS. Cell 2022: PMID: 36055201. Is there a specific reason that they did not use that?*

We use “1000 Genomes Project” through the revised paper.

Phase 3 version of 1000 Genomes Project data were used in the study. The reference to the project is updated.

*Have they examined the allele frequency distribution of variants on the X vs autosomes in dbSNP?*

No, we did not do this specific analysis. The reason for not pursuing this analysis is that including SNP frequencies into the analysis would add a new level of complexity and would make the study less focused. There is a significant variation in allele frequencies between populations suggesting that we would need to use population-specific GWAS hits. Many GWA studies include different populations in analysis. Our goal was to detect X-chromosome versus autosome differences that can be detected by pan-population analysis.

*In addition to GWAS of female-specific traits, there have been some GWAS of traits in sex-specific fashion (e.g. WHR). Could this add useful information?*

For female-only analysis we used GWASs targeting female specific phenotypes. We did not use traits that can be measured in both sexes like waist-to-hip ratio. Even though these traits may be present in both sexes, sex-specific analyses may reflect sexual dimorphism and therefore the interpretation of findings from these studies may be complex. We therefore have not chosen to include them.

*How do you know that these female-specific GWASes even analysed the X chromosome?*

We did not check if female-specific GWASs included X-chromosomal SNPs. Because these studies used standard genotyping platforms that included X-chromosome SNPs, we assumed that female-restricted GWASs targeted X-chromosome SNPs.

*Regarding call rate, there is a typo in the ‘Affymetrix platRform’ in Fig 6. Can the authors be specific about which Affy platforms, since some had very high rates of variants being cleaned due to various QC issues, e.g. https://www.ncbi.nlm.nih.gov/pmc/articles/PMC4072456/ Also, they do not provide X chr call rates stratified by sex. This is important cince call rate is a common QC metric.*

We fixed the typo on Fig. 6.

We used Affymetrix Axiom array in the analysis. We indicated this in the revision.

We provide X-chromosome call rates stratified by sex: blue bars – males, red – females.

*Regarding HWE, there is a more important issue in females on X that one generation of random mating is not sufficient to produce HWE in females – it can require many more generations. I think Graffelman & Weir discussed this (Heredity, 2016) who cite Kimura and Crow 1970. Is there any information about what % of SNPs are failing HWE on X vs autosomes?*

We added the following paragraph to the Discussion: “X-chromosome SNPs do not show a higher deviation from HWE compared to autosomal SNPs. In fact, Graffelman et al. (2017) [17] found that deviation from HWE is higher for autosomal SNPs. “

*For the analysis of SNP density (Intergenic/intronic/exonic), the authors should acknowledge that phase 3 1000 genomes used high coverage WES (including many different heterogeneous capture and sequencing technologies), but only low coverage WGS (in addition to two GWAS arrays). It would be interesting to see if this difference exists for the recent high coverage WGS from 1000 Genomes (see above). In addition, since there are both synonymous and non-synonymous exonic variants, do they see differences in density between these groups? It would be helpful to label the X axes of the Fig 7 by chromosome number. What happens when they look in ClinVar variants, does that support fewer bioinformatically predicted functional variants on X genes than autosomes?*

We acknowledged lower coverage of intergenic regions in 1000 Genomes Project phase 3 data.

We indicated that the positions of chromosomes on the Figure 7 from left to right correspond to chromosomal numbers.

We do not think that the comparison of proportions of different types of mutations within exonic regions is informative. Estimating proportions of segregating sites is a better approach for comparative analysis of purifying selection. The percentage of segregating sites among all potential sites can be used for a comparative analysis of purifying selection because the number of potential sites on a chromosome is fixed. In the revision we used the proportion of segregating sites in autosomal and X-chromosome genes for missense mutations categorized by bioinformatics prediction of functional effect.

The following text was added:

“Since the majority of *de novo* mutations are deleterious [18], purifying form of selection is the most prevalent [19]. Purifying selection against deleterious mutations may change frequencies of the linked SNPs. This form of the linked selection is called background selection [20-22]. Background selection is widespread and significantly influences evolutionary dynamics of the linked SNPs regardless of their functional significance [22, 23]. Therefore, changes in frequency of functional polymorphisms (e.g. missense mutations) usually change frequencies of linked nonfunctional SNPs (e.g. synonymous SNPs) making it challenging to compare selection by using proportions of different types of exonic SNPs in X-chromosome and autosomes.

We used Envision bioinformatics tool to predict the effect of missense mutations on protein function [24]. Envision combines experimental data on functional effects of missense mutations with a supervised, stochastic gradient boosting learning algorithm to quantify functional effects of missense mutations. Envision outperforms similar bioinformatics tools for prediction of functional effects [24]. Envision provides precomputed predictions for every possible single amino acid substitution in the human proteome. Among all possible amino acid substitutions (19 possible substations per amino acid) we identified the substitutions that can be produced by a single nucleotide substitution (SNS). For this we computationally mutated each single nucleotide in the coding sequence into 3 possible SNSs and identified those producing missense mutations (see [25] for details). Possible missense mutations were stratified into 7 categories based on the Envision score – the lower score is associated with a stronger functional disturbance. Therefore, for each gene we estimated the number of potential sites for each category. We used 1000 Genomes Project data to identify how many of the potential sites exits as SNPs (Supplementary Table S5).

The ratio of segregating sites to the number of potential sites was used as a measure of purifying selection [26]. Sites under strong selection are expected to be less polymorphic. The results of the analysis stratified by chromosome are shown on Figure 8. Proportions of segregating sites increase with a higher Envision score. There are striking differences in the proportion of segregating sites between X-chromosome and autosomes: X-chromosome has a consistently lower proportion of segregating sites compare to autosomes. This is consistent with the idea that selection against X-chromosome missense mutations is stronger compared to autosomal missenses mutations.

The analysis of proportions of segregating sites for the 7 categories of missense mutations stratified by the predicted effect on the protein function demonstrated that (i) the proportion of segregating sites increases as the predicted functional effect of missense mutations decreases for all chromosomes, and that (ii) the proportion of segregating sites for X-chromosome mutations is lower than the proportion of segregating sites for missense mutations in autosomal genes. The results provide support for the hypothesis of lower density of functional polymorphisms on X-chromosome as a result of stronger purifying selection.

**Figure 8**. The proportion of segregating sites for missense mutations categorized by Envision score. The lower envision score is associated with a stronger effect on protein function. Of note, there is a higher inter chromosomal variation in the proportions of segregating sites on the tails of the distribution because of small sample sizes for those categories (see Supplementary Table S5)

*Is the gene density similar on X vs autosomes?*

Roughly “yes”: the number of genes per 100,000 nucleotides equals 1.44±0.13 for autosomes and 1.23 for X-chromosome.

*‘Twice lower’ would be better worded as half’.*
We changed the wording.

*When they argue that for a dominant model the sample size is small for males may be true, but most analysis assumes additive model. What is the implication on power then?*

We removed the confusing reference to the dominant model from the text.

*Analysis of density across the X chromosome assumes that there are no gaps in the sequence. My recollection is that particularly in PAR1 there are multiple large gaps in GRCh37, which typically would result in low SNP density and poor imputation (if even attempted or possible). What happens when they take such gaps into account? Do they see reflections of these gaps when they look at the density of SNPs on different arrays? A recent paper examined population genetics of PARs and may contain relevant information: PMID:33872316.*

Since we estimated the fraction of GWAS-detected SNPs among SNPs in the region rather than size of the region it is unlikely that SNP detection and imputation success play major role in the density of GWAS hits in the region. Yet we added a cautionary note and cited PMID:33872316 paper in the revision.

*I have a similar comment related to gaps in sequence in other chromosomes. For example the first ~ 5Mb of chr 21 has N in GRCh18. Did they count this to the genic region in Tables S1?*

Yes, ambiguously sequenced sites were included when the total chromosome size was estimated.

*How were sequential meta-analysis of the same trait dealt with in the analysis. For example there are 268 studies of BMI in the GWAS catalog today. Presumably most found the loci with largest effect (e.g. FTO). Are they counting these as independent or a single signal?*

SNPs detected and reported by multiple GWAS were counted as a single signal even when it was associated with different phenotypes or diseases. We added the above statement to the revision.

***Have all data underlying the figures and results presented in the manuscript been provided?*** *Large-scale datasets should be made available via a public repository as described in the PLOS Genetics* [*data availability policy*](https://urldefense.proofpoint.com/v2/url?u=http-3A__journals.plos.org_plosgenetics_s_data-2Davailability&d=DwMGaQ&c=ZQs-KZ8oxEw0p81sqgiaRA&r=rBUX5B9Uo388Kv5SGxaplHD76qSm4u7_TQ3ohcupaCQ&m=v-RgtedOrFebQL8kvHHaoQt9lccZfR84Y9g2bVuoQN_2N-WXPbUDrCsH7ITodNht&s=bEXHmqy6-Ro0TAoGzGmT79TbJ6_WoDGZ6L_y3cp-ZwY&e=)*, and numerical data that underlies graphs or summary statistics should be provided in spreadsheet form as supporting information.*

*Reviewer #1: Yes*

*Reviewer #2:* ***No:*** *They say they reviewed 20 random GWASes, but do not provide the references for them.*

We provided the list of randomly selected GWASes we have used (Supplementary Table S5).

1.	Purcell S, Neale B, Todd-Brown K, Thomas L, Ferreira MA, Bender D, et al. PLINK: a tool set for whole-genome association and population-based linkage analyses. Am J Hum Genet. 2007;81(3):559-75. Epub 2007/08/19. doi: 10.1086/519795. PubMed PMID: 17701901; PubMed Central PMCID: PMCPMC1950838.

2.	Konig IR, Loley C, Erdmann J, Ziegler A. How to include chromosome X in your genome-wide association study. Genet Epidemiol. 2014;38(2):97-103. Epub 2014/01/11. doi: 10.1002/gepi.21782. PubMed PMID: 24408308.

3.	Gogarten SM, Bhangale T, Conomos MP, Laurie CA, McHugh CP, Painter I, et al. GWASTools: an R/Bioconductor package for quality control and analysis of genome-wide association studies. Bioinformatics. 2012;28(24):3329-31. Epub 2012/10/12. doi: 10.1093/bioinformatics/bts610. PubMed PMID: 23052040; PubMed Central PMCID: PMCPMC3519456.

4.	Graffelman J, Weir BS. Testing for Hardy-Weinberg equilibrium at biallelic genetic markers on the X chromosome. Heredity (Edinb). 2016;116(6):558-68. Epub 2016/04/14. doi: 10.1038/hdy.2016.20. PubMed PMID: 27071844; PubMed Central PMCID: PMCPMC4868269.

5.	Huentelman MJ, Craig DW, Shieh AD, Corneveaux JJ, Hu-Lince D, Pearson JV, et al. SNiPer: improved SNP genotype calling for Affymetrix 10K GeneChip microarray data. BMC Genomics. 2005;6:149. Epub 2005/11/03. doi: 10.1186/1471-2164-6-149. PubMed PMID: 16262895; PubMed Central PMCID: PMCPMC1280925.

6.	Bertolini F, Elbeltagy AR, Rothschild MF. Evaluation of the application of bovine, ovine and caprine SNP chips to dromedary genotyping. Livestock research for rural development. 2017;29:31.

7.	Sachidanandam R, Weissman D, Schmidt SC, Kakol JM, Stein LD, Marth G, et al. A map of human genome sequence variation containing 1.42 million single nucleotide polymorphisms. Nature. 2001;409(6822):928-33. Epub 2001/03/10. doi: 10.1038/35057149. PubMed PMID: 11237013.

8.	Wilson Sayres MA. Genetic Diversity on the Sex Chromosomes. Genome Biol Evol. 2018;10(4):1064-78. Epub 2018/04/11. doi: 10.1093/gbe/evy039. PubMed PMID: 29635328; PubMed Central PMCID: PMCPMC5892150.

9.	Arbiza L, Gottipati S, Siepel A, Keinan A. Contrasting X-linked and autosomal diversity across 14 human populations. Am J Hum Genet. 2014;94(6):827-44. Epub 2014/05/20. doi: 10.1016/j.ajhg.2014.04.011. PubMed PMID: 24836452; PubMed Central PMCID: PMCPMC4121480.

10.	Naj AC. Genotype Imputation in Genome-Wide Association Studies. Curr Protoc Hum Genet. 2019;102(1):e84. Epub 2019/06/20. doi: 10.1002/cphg.84. PubMed PMID: 31216114.

11.	Porcu E, Sanna S, Fuchsberger C, Fritsche LG. Genotype imputation in genome-wide association studies. Curr Protoc Hum Genet. 2013;Chapter 1:Unit 1 25. Epub 2013/07/16. doi: 10.1002/0471142905.hg0125s78. PubMed PMID: 23853078.

12.	Schurz H, Muller SJ, van Helden PD, Tromp G, Hoal EG, Kinnear CJ, et al. Evaluating the Accuracy of Imputation Methods in a Five-Way Admixed Population. Front Genet. 2019;10:34. Epub 2019/02/26. doi: 10.3389/fgene.2019.00034. PubMed PMID: 30804980; PubMed Central PMCID: PMCPMC6370942.

13.	Panagiotou OA, Willer CJ, Hirschhorn JN, Ioannidis JP. The power of meta-analysis in genome-wide association studies. Annu Rev Genomics Hum Genet. 2013;14:441-65. Epub 2013/06/04. doi: 10.1146/annurev-genom-091212-153520. PubMed PMID: 23724904; PubMed Central PMCID: PMCPMC4040957.

14.	Zeggini E, Ioannidis JP. Meta-analysis in genome-wide association studies. Pharmacogenomics. 2009;10(2):191-201. Epub 2009/02/12. doi: 10.2217/14622416.10.2.191. PubMed PMID: 19207020; PubMed Central PMCID: PMCPMC2695132.

15.	Wang Z, Sun L, Paterson AD. Major sex differences in allele frequencies for X chromosomal variants in both the 1000 Genomes Project and gnomAD. PLoS Genet. 2022;18(5):e1010231. Epub 2022/06/01. doi: 10.1371/journal.pgen.1010231. PubMed PMID: 35639794; PubMed Central PMCID: PMCPMC9187127.

16.	Vuckovic D, Bao EL, Akbari P, Lareau CA, Mousas A, Jiang T, et al. The Polygenic and Monogenic Basis of Blood Traits and Diseases. Cell. 2020;182(5):1214-31 e11. Epub 2020/09/06. doi: 10.1016/j.cell.2020.08.008. PubMed PMID: 32888494; PubMed Central PMCID: PMCPMC7482360.

17.	Graffelman J, Jain D, Weir B. A genome-wide study of Hardy-Weinberg equilibrium with next generation sequence data. Human genetics. 2017;136(6):727-41. Epub 2017/04/05. doi: 10.1007/s00439-017-1786-7. PubMed PMID: 28374190; PubMed Central PMCID: PMCPMC5429372 conflict of interest.

18.	Keightley PD. Rates and fitness consequences of new mutations in humans. Genetics. 2012;190(2):295-304. Epub 2012/02/22. doi: 10.1534/genetics.111.134668. PubMed PMID: 22345605; PubMed Central PMCID: PMCPMC3276617.

19.	Hughes AL, Packer B, Welch R, Bergen AW, Chanock SJ, Yeager M. Widespread purifying selection at polymorphic sites in human protein-coding loci. Proc Natl Acad Sci U S A. 2003;100(26):15754-7. Epub 2003/12/09. doi: 10.1073/pnas.2536718100. PubMed PMID: 14660790; PubMed Central PMCID: PMCPMC307640.

20.	Stephan W. Genetic hitchhiking versus background selection: the controversy and its implications. Philos Trans R Soc Lond B Biol Sci. 2010;365(1544):1245-53. Epub 2010/03/24. doi: 10.1098/rstb.2009.0278. PubMed PMID: 20308100; PubMed Central PMCID: PMCPMC2871815.

21.	Hodgkinson A, Casals F, Idaghdour Y, Grenier JC, Hernandez RD, Awadalla P. Selective constraint, background selection, and mutation accumulation variability within and between human populations. BMC Genomics. 2013;14:495. Epub 2013/07/24. doi: 10.1186/1471-2164-14-495. PubMed PMID: 23875710; PubMed Central PMCID: PMCPMC3727949.

22.	Charlesworth B. Background selection 20 years on: the Wilhelmine E. Key 2012 invitational lecture. J Hered. 2013;104(2):161-71. Epub 2013/01/11. doi: 10.1093/jhered/ess136. PubMed PMID: 23303522.

23.	Wright SI. Charlesworth et al. on Background Selection and Neutral Diversity. Genetics. 2016;204(3):829-32. Epub 2017/01/24. doi: 10.1534/genetics.116.196170. PubMed PMID: 28114095; PubMed Central PMCID: PMCPMC5105860.

24.	Gray VE, Hause RJ, Luebeck J, Shendure J, Fowler DM. Quantitative Missense Variant Effect Prediction Using Large-Scale Mutagenesis Data. Cell Syst. 2018;6(1):116-24 e3. Epub 2017/12/12. doi: 10.1016/j.cels.2017.11.003. PubMed PMID: 29226803; PubMed Central PMCID: PMCPMC5799033.

25.	Gorlova Olga KM, Tsavachidis Spiridon, Amos Christopher, Gorlov Ivan. Identification of lung cancer drivers by comparison of the observed and the expected numbers of missense and nonsense mutations in individual human genes. Oncotarget. 2022;V14(1):17-29.

26.	Zivkovic D, Wiehe T. Second-order moments of segregating sites under variable population size. Genetics. 2008;180(1):341-57. Epub 2008/08/22. doi: 10.1534/genetics.108.091231. PubMed PMID: 18716326; PubMed Central PMCID: PMCPMC2535686.
