# Supplementary material for: Why does the X chromosome lag behind autosomes in GWAS findings?
Source: PLoS Genet. 2023 Feb 27;19(2):e1010472. doi: 10.1371/journal.pgen.1010472 (PMC9997976; doi:10.1371/journal.pgen.1010472)
Supplement: Attachment — Submitted filename: Response to Reviwers_PLoS_Genet_X-chrc_a.docx [file pgen.1010472.s008.docx]

Dear Drs. Ziegler and Williams,

We are resubmitting a revised paper, '**Why does the X chromosome lag behind autosomes in GWAS findings?**' We provide point-by-point responses to the Reviewers’ comments. All corrections in the revised manuscript are shown in red.

***Reviewer #1: The authors have addressed my concerns.***

***Reviewer #2:***

*In general, the authors provide adequate responses to my previous comments. However, they do not provide any justification for their use of the phase 3 1000 genomes data rather than the high coverage data (https://www.sciencedirect.com/science/article/pii/S0092867422009916). In particular, there were many different capture and sequencing technologies used for the exome sequencing in phase 3, that could in part be driving the apparent paucity of bioinformatically predicted functional variants on X (which of course tend to be rare and are most impacted by technical errors). This is puzzling, since the high coverage data is of much higher quality. Recently, the high-coverage data has been re-aligned to the T2T-CHM13 consortium build, in a Science paper entitled ‘A complete reference genome IMPROVES analysis of human genetic variation‘ (https://www.science.org/doi/10.1126/science.abl3533), making it even more puzzling why they persist with the phase 3 data.*

When we started this analysis more than two years ago 1,000 genomes and TOPMed SNP data were largest publically available data sources on whole-genome detected SNPs we were aware about. Papers mentioned by the Reviewer were published in 2022 when our analysis was completed and we were in the process of finalizing the manuscript. T2T-CHM13 human genome build when used as a reference genome added over a million SNPs to 1000 genomes, which is impressive in absolute value but comprises only about 1% of 1000 genome SNPs, making it unlikely to change the major conclusions of the study. To date the data deposited from this consortium (https://www.ncbi.nlm.nih.gov/assembly/GCA_009914755.4) comprises only 24 chromosomes, so the reliability of the SNP allele frequencies that derive from the consortium remains low.

*Fig 7. Still this figure has ‘one thousand genome SNPs’ as title. Also, TopMED should be TOPMed.*

We corrected Fig. 7.

*The authors should describe the limitations of using data from dbSNP since it is a user- submitted database. For example it has been shown that conventional bioinformatic approaches to variant calling on the sex chromosomes results in failure to call thousands of variants per subject (PMID: 31289836), especially in the PARs, and it is my impression that few (or none) of the consortia that submit large numbers of SNPs to dbSNP have employed this modified method. The authors make no mention of this, and the problem is potentially even worse for 1000 genomes phase 3 data, since I remember that they performed an ensemble variant calling across ~20 different methods, and although I have not examined each and every method, I am skeptical that any of them employed X-specific variant calling.*

The following sentence was added to the part discussing limitations of dbSNP database: “Also because the data provided to the dbSNP database are submitted by individual users, in some cases this can lead to a bias against X-chromosome SNPs when the authors use inadequate variant calling methods [1].”

*The authors respond to the HWE question, by referring to Graffelman 2016 Fig 7. This figure shows qq plots for females and males for chi2 and exact tests. There are no direct comparisons for results across SNPs, so it is not correct for the authors to make any statement that ‘For some SNPs the results … are different,… but overall are not substantial’. Although Graffelman 2016 state the inflation of the chi2 is due to rare alleles, there is no data shown to support this conclusion. Further, in the Discussion when the authors cite Graffelman 2017 comparing autosomes to X for HWE, they should be clear that these data come again from 1000 Genomes phase 3, from two very small populations, and the limitations of which I have described above (e.g. mean depth=6).*

A note of caution was added to the sentence citing the paper by Graffelman et al. (2017): “In fact, Graffelman et al. (2017) [2] found that the deviation from HWE is higher for autosomal SNPs. This finding, however, needs to be taken with caution because the study was based on the data from 1000 Genomes samples with a relatively low sequencing depth.”

*When the authors analyse TOPMed data, they should consider the nature of the cohorts that contribute to TOPMed, and the bias that this may introduce. As I understand this is an NHLBI-funded program, including classic CVD cohorts. I have never seen the age distribution of TOPMed as a whole, but I suspect it’s relatively old. Could loss of X diversity due to premature mortality from XLR diseases result in part of the apparent deficit of X exonic variants. Is there a birth cohort that could be used for comparison purposes? For example, variants in COL4A5 (Xq22.3) are a common cause of Alport syndrome, that classically presents in childhood/early adulthood in males. Individuals with Alport syndrome due to variants in this gene typically develop end-stage kidney disease in their 30s and likely either are not alive to participate in cohorts like those in TOPMed, or are too sick to want to participate. It would be useful to look at common severe XLR disease genes (FRAX, DMD, BMD,. hemophilia A, G6PD) to see whether the diversity in those genes differs from other X genes. Similar considerations likely apply to 1000 Genomes samples: I presume that only adults were approached to be included.*

“*Premature mortality from XLR diseases*” cannot explain “*the apparent deficit of X exonic variants.”* Population prevalence of X-Linked Recessive (XLR) disease is low:

1 in every 50,000 live births for Alport syndrome;

1 per 170,000 for Hunter’s disease

1 per 250,000 for Anhidrotic ectodermal dysplasia

1 per 250,000 for Dyskeratosis congenita

1 per 50,000 for SCID

1 per 1,000,000 for Wiskott-Aldrich syndrome

1 per 100,000 for X-linked Fabry’s disease

1 per 10,000 for FXS syndrome

1 per 100,000 for Ehlers-Danlos syndrome

1 per 380,000 for Lesch-Nyhan syndrome

Population prevalence of G6PD deficiency is about 3%. Typically this condition is not life threatening and the absolute majority of people remain clinically asymptomatic throughout their lives. Even if we assume that all XLR variants carrier “*are not alive to participate in cohorts like those in TOPMed”* the expected selection is not enough to explain the observed 6 fold differences in the density of GWAS-detected SNPs between X-chromosome and autosomes.

Since age is such a strong predictor of diseases, it is a standard practice to match cases and controls by age. Therefore cases and controls in GWASs targeting XLR diseases are expected to be matched by age which argues against “premature mortality from XLR diseases” as a reason for the striking deficit of GWAS detected SNPs on X-chromosome.

As the Reviewer advised we compared densities of TOPMed SNPs in genes associated with common severe XLR disease genes: *FRAX, DMD, F8*, and *G6PD* with overall densities of SNPs in X-chromosomal genes. Exonic and intronic SNPs were analyzed separately. We found no significant difference in SNP densities (number of TOPMed SNPs per 100 nucleotide) between XLR disease genes compared to other genes on X-chromosome: 13.4±2.1 versus 12.2±0.2 for intronic and 12.6±2.0 versus 11.9±0.2 for exonic SNPs. The result suggests that selection against SNPs linked to XLR disease is unlikely to drive the profound differences in the density of GWAS-detected SNPs between X-chromosome and autosomes.

1.	Webster TH, Couse M, Grande BM, Karlins E, Phung TN, Richmond PA, et al. Identifying, understanding, and correcting technical artifacts on the sex chromosomes in next-generation sequencing data. Gigascience. 2019;8(7). Epub 2019/07/11. doi: 10.1093/gigascience/giz074. PubMed PMID: 31289836; PubMed Central PMCID: PMCPMC6615978.

2.	Graffelman J, Jain D, Weir B. A genome-wide study of Hardy-Weinberg equilibrium with next generation sequence data. Human genetics. 2017;136(6):727-41. Epub 2017/04/05. doi: 10.1007/s00439-017-1786-7. PubMed PMID: 28374190; PubMed Central PMCID: PMCPMC5429372 conflict of interest.
